# Quantitative Proteomic Analysis of Duck Embryo Fibroblasts Infected With Novel Duck Reovirus

**DOI:** 10.3389/fvets.2020.577370

**Published:** 2020-12-02

**Authors:** Yudong Yang, Lin Li, Xingpo Liu, Meijie Jiang, Jun Zhao, Xuesong Li, Cui Zhao, Hui Yi, Sidang Liu, Ning Li

**Affiliations:** ^1^Shandong Provincial Key Laboratory of Animal Biotechnology and Disease Control and Prevention, Shandong Provincial Engineering Technology Research Center of Animal Disease Control and Prevention, Sino-German Cooperative Research Centre for Zoonosis of Animal Origin Shandong Province, College of Animal Science and Technology, Shandong Agricultural University, Taian, China; ^2^Taian City Central Hospital, Taian, China; ^3^Shanghai Veterinary Research Institute, Chinese Academy of Agricultural Sciences, Shanghai, China; ^4^Taian City Animal Husbandry and Veterinary Service Center, Taian, China

**Keywords:** novel duck reovirus, proteomic analysis, cellular response, interferon-stimulated genes, iTRAQ

## Abstract

The novel duck reovirus (NDRV) can cause hemorrhage and necrosis on the spleen of Pekin ducks; this disease has resulted in great economic losses to the duck industry. However, the molecular pathogenesis of NDRV remains poorly understood. In the current study, the quantitative proteomic analysis of NDRV-infected duck embryo fibroblasts was performed to explore the cellular protein changes in response to viral infection through iTRAQ coupled with the liquid chromatography (LC)–tandem mass spectrometry (MS/MS) method. A total of 6,137 proteins were obtained in cell samples at 24 h post-infection. Of these, 179 differentially expressed proteins (DEPs) were identified (cutoff set to 1.5-fold change), including 89 upregulated and 90 downregulated proteins. Bioinformatics analysis showed that DEPs can be divided into the cellular component, molecular function, and biological process; they were mainly involved in signal transduction, infectious diseases, cell growth and death, and the immune system. The subcellular localization of most proteins was in the cytoplasm. Importantly, the expressions of signal transducer and activator of transcription 1 (STAT1) and various interferon-stimulated genes (ISGs) were upregulated after NDRV infection. The mRNA transcripts of some ISGs were consistent with proteomic data, showing an increased trend. Results of our study suggested that NDRV infection can elicit strong expression changes of cellular proteins and activate the expression of ISGs from the point of quantitative proteomic analysis. The study provides a new insight into the understanding of NDRV pathogenesis.

## Introduction

Novel duck reovirus (NDRV) is a member of the genus *Orthoreovirus*, belonging to the family *Reoviridae*. The virus contains a segmented double-stranded RNA genome with 10 segments, which can be divided into three groups: large (L1–L3), medium (M1–M3), and small (S1–S4) segments, according to the mobility of the genes on gel electrophoresis. The genome of NDRV encodes at least 10 structural and four nonstructural proteins (1). The infectious disease caused by NDRV was first reported in several southern provinces of China in 2006; various breeds of ducks can be infected, including Pekin duck, Cherry Valley duck, shelduck, Muscovy duck, etc. The disease has caused great economic losses to the duck industry since its outbreak (2, 3).

The pathological changes of this disease are related to the duck breed. The main manifestations of NDRV-infected Muscovy duck and shelduck are hemorrhage and necrosis of the liver, splenomegaly with necrotic foci, and hemorrhage of the kidney and bursa of Fabricius (4, 5). However, the Pekin duck and Cherry Valley duck infected with NDRV are mainly characterized by hemorrhage and necrosis of the spleen (3, 6). Moreover, the immunosuppression caused by the NDRV infection increases the susceptibility to concurrent or secondary bacterial or viral infections. The phylogenetic analysis shows that NDRV strains are genotype II, which is different from the classical Muscovy duck reovirus (MDRV) strains belonging to genotype I. The nucleotide homology of most genes between NDRV and MDRV is low, especially the σC protein encoding by S1 gene is only 36.3% (1), indicating that there is a significant difference between these two types of viruses, namely, NDRV is different from MDRV. Therefore, the pathogenesis of NDRV may be different from the classical MDRV, and this difference needs further investigation.

The complex interaction between virus and host is critical for understanding the pathogenesis of viral infection. It is well-recognized that transcriptomic and proteomic analyses are good and effective methods for large-scale screening of the host genes and proteins after a viral infection. In MDRV infection experiments *in vivo* and *in vitro*, transcriptomic and proteomic analyses demonstrated a large number of differentially expressed genes and proteins (DEPs), which were involved in multiple biological processes, such as the regulation of immune and cellular processes and metabolism (7, 8). However, few proteomic analyses regarding NDRV infection have been conducted. Recently, two studies (9, 10) compared the proteomic results in the liver and spleen of NDRV-infected Muscovy ducks with those of MDRV-infected ducks and found that the host response patterns to the MDRV and NRDV infections might be tissue-dependent. Only a small number of common DEPs in both organs were identified, and the serine protease systems were activated in both the liver and spleen, indicating that the serine protease-mediated innate immunity might play an important role in the defense against reovirus infection (10). Considering the diversity of duck breeds and the limitation of reproducibility of experiments *in vivo*, some studies were conducted in cells. Duck Tembusu virus, the emerging duck-origin flavivirus (11), infected BHK-21 cells, then quantitative proteomics were used to identify DEPs, and the results showed that many innate immune-related proteins were involved in viral replication (12). Nevertheless, the information about duck-origin cell responses to NDRV infection is absent.

In this study, iTRAQ combined with liquid chromatography (LC)–tandem mass spectrometry (MS/MS) analysis approach was used to identify the protein profiles of NDRV-infected duck embryo fibroblasts (DEFs). At 24 h after NDRV infection, a total of 179 proteins were upregulated (89) or downregulated (90) (cutoff is 1.5-fold change) in either the nuclear or the cytoplasmic fractions. Further bioinformatics analysis revealed that these DEPs were implicated in several biological systems and processes. Notably, the expression of many interferon (IFN)-stimulated genes (ISGs) was significantly upregulated. This information will provide a better understanding of the interaction between the pathogenesis of NDRV and the host immune response and may provide insight into the development of potential antiviral agents.

## Materials and Methods

### Cells and Virus

DEFs were produced using 10-day-old duck embryos in the experiment and were grown in Dulbecco's modified Eagle's medium (DMEM) (GIBCO, Grand Island, NY, USA) supplemented with 10% fetal bovine serum (GIBCO) at 37°C and 5% CO_2_. The NDRV strain used in this study was isolated from clinically infected duck and stored in our laboratory (6). The virus was propagated in BHK-21 cells, and the titer was determined to be 2 × 10^4.6^ tissue culture infectious dose (TCID_50_)/ml in DEF cells.

### Virus Infection

DEF cells were passaged before infection and were seeded in six-well-plates (Corning, Shanghai, China) with 2.5 × 10^6^ cells per well. The monolayer cells were inoculated with NDRV (2 × 10^2.6^ TCID_50_/well). After incubation for 1 h, the virus solution was removed and the cells were washed with sterile phosphate buffered saline. The DMEM containing 1% serum was added, and the cells were maintained at 37°C and 5% CO_2_. Negative control cultures were set up identically but incubated with serum-free DMEM. The cells were collected for protein isolation, digestion, and labeling at 24 h post-infection (hpi). The propagation of NDRV was analyzed by observation of the cytopathic effect (CPE) and the one-step growth curve in DEF cells at the indicated time points by quantitative PCR (qPCR) (6). Each group included three independent biological replicates.

### Protein Extraction, Digestion, and iTRAQ Labeling

The cells of the infected group and the control group were collected and resuspended in 500 μl of lysis buffer (7 M urea, 2 M thiourea, 20 mM Tris, and pH 8–8.5) containing 1 mM phenylmethylsulfonyl fluoride (PMSF) and incubated on ice for 5 min. Cell samples were ultrasonicated on ice for 2 min (50 Hz) with cellular debris removed by centrifugation (25,000 × g, 15 min, 4°C). The supernatant containing 10 mM dithiothreitol (DTT) with a final concentration of 4% was treated in water baths at 56°C for 1 h. Then, the iodoacetamide (IAM) with a final concentration of 55 mM was added and placed in a dark room for 45 min. The protein samples were harvested by centrifugation at 25,000 × g for 20 min at 4°C. The protein concentration of the supernatant was determined with the Bradford method (13). Solutions containing 100 μg protein were digested for 12 h at 37°C with sequencing-grade trypsin and then labeled with different iTRAQ tags as follows: the three negative samples were each labeled with iTRAQ 118 (IT118), IT119, or IT121; and the three NDRV-infected samples were each labeled with IT115, IT116, or IT117. The labeled samples were incubated at room temperature for 2 h then mixed, and dried with a rotary vacuum concentrator.

### Liquid Chromatography–Tandem Mass Spectrometry Analysis

The LC-20AB liquid-phase system (Shimadzu, Kyoto, Japan) was used, and the separation column was a 5 μm 4.6 × 250 mm Gemini C18 column for liquid-phase separation of samples. The pumped peptide sample with mobile phase A [5% acetonitrile (ACN) pH 9.8] was reconstituted and injected and eluted at a flow rate gradient of 1 ml/min: 5% mobile phase B (95% ACN, pH 9.8) for 10 min, 5–35% mobile phase B for 40 min, 35–95% mobile phase B for 1 min. The mobile phase B lasted 3 min, and the 5% mobile phase B was equilibrated for 10 min. The elution peak was monitored at a wavelength of 214 nm, and one component was collected every minute. The sample was combined with the chromatographic elution peak to obtain 20 components and then freeze-dried. The dried peptide sample was reconstituted with mobile phase A (2% ACN, 0.1% FA) and centrifuged at 20,000 × g for 10 min, and the supernatant was injected. Separation was performed by UltiMate 3000 UHPLC (Thermo Fisher Scientific, San Jose, CA, USA). The sample first entered a trap column for enrichment and desalting and was then connected in series with a self-packed C18 column (75 μm inner diameter, 3 μm column > particle size, and 25 μm column length) and separated at a flow rate of 300 nl/min through the following effective gradient: 0–5 min and 5% mobile phase B [98% ACN, 0.1% formic acid (FA)] 5–45 min. The mobile phase B linearly increased from 5 to 25%, 45–50 min, the mobile phase B increased from 25 to 35%, 50–52 min, the mobile phase B increased from 35 to 80%, 52–54 min, the 80% mobile phase B; 54–60 min, 5% mobile phase B. The nanoliter liquid separation end was directly connected to the mass spectrometer.

The peptides separated by the liquid phase were ionized by the nanoESI source and entered the tandem mass spectrometer Q-Exactive HF X (Thermo Fisher Scientific, San Jose, CA, USA) for data-dependent acquisition mode detection. Main parameter settings: the ion source voltage was set to 1.9 kV, the scanning range of the primary spectrum was 350–1,500 m/z, the resolution was set to 60,000, the starting m/z of the secondary spectrum was fixed to 100, the resolution was 15,000. The screening conditions for the second-stage fragmented precursor ions were charge 2+ to 6+, and peak intensities exceeding 10,000 were ranked in the top 20 precursor ions. The ion fragmentation mode was higher-energy collisional dissociation (HCD), and the fragment ions were detected in Orbitrap (Thermo Fisher Scientific, San Jose, CA, USA). The dynamic exclusion time was set to 30 s. The automatic gain control (AGC) was set to: level 1 3E6, level 2 1E5.

### Protein Identification and Quantification

Mascot software (Matrix Science, London, UK) was used to search and identify the processed mass spectrometer offline data. In bioinformatics analysis, the original mass spectrum file needs to be converted into mascot generic format (MGF) before it can be used. The MGF file mainly contains the information of the secondary MS spectrum, and then the converted file is searched by the identification Mascot software and the selected protein sequence database (Uniprot–*Anas platyrhynchos*) to obtain the final protein identification result. The selected trusted protein must contain at least one trusted unique peptide.

For the quantification of iTRAQ data, we used the iQuant software independently developed by Huada, which integrates the Mascot Percolator algorithm (12), which uses machine learning algorithms to automatically re-score database search results, thereby improving the identification rate of results. First, 1% false discovery rate (FDR) filtering was performed at the spectrum/peptide level (PSM-level FDR ≤0.01) to obtain significantly identified spectra and peptide lists. Then, based on the “parsimony principle,” peptide assembly was used to produce a series of proteomes. In order to control the false-positive rate of the protein, the process was filtered again at the protein level with FDR 1% (protein-level FDR ≤0.01). The strategy for the picked protein FDR using iQuant mainly includes the following steps: protein filtration, report-group tag-purity correction, quantitative value normalization, missing value completion, protein quantitative-value calculation, statistical test analysis, and final result display.

### Bioinformatics Analysis

In the Gene Ontology (GO) analysis (http://www.geneontology.org/), by comparing the identified proteins with the NR database (RefSeq non-redundant proteins), the corresponding GO functional classification was obtained. Pathway classification is to directly compare the identified protein sequences with the Kyoto Encyclopedia of Genes and Genomes (KEGG) database through Blast software (http://www.genome.jp/kegg) to obtain corresponding annotation information and complete pathway classification results. WoLF PSORT (https://www.genscript.com/wolf-psort.html) is used to predict protein subcellular localization of eukaryotes.

### RNA Extraction and Quantitative Real-Time PCR

Total RNA of cells collected at 24 hpi was extracted using the TRIzol reagent (Takara, Dalian, China) according to the manufacturer's instructions (14). One microgram RNA was reverse-transcribed to cDNA by ReverTra AceR qPCR RT Master Mix with gDNA Remover kit (Toyobo Co., Ltd., Osaka, Japan). The primers used in this study were referred from other reports (15–17). qPCR was performed using the TB Green™ Fast qPCR Mix (Takara, Dalian, China) on Roche LightCycler 96 (Roche, Basel, Switzerland). The total qPCR reaction volume contains 10 μl 2× TB Green Premix Ex Taq II (Takara, Dalian, China): 1 μl template cDNA, 0.8 μl of each forward and reverse primer (10 μM), and in a final volume of 20 μl. The qPCR conditions were as follows: one cycle at 95°C for 30 s, followed by 40 cycles at 95°C for 5 s, and 60°C for 30 s. The melting curve was analyzed for confirming specific amplification. Three independent assays were performed with each sample analyzed in triplicate.

### Statistical Analysis

The housekeeping gene β-actin was used as an internal control to normalize the mRNA expression, and the relative fold changes of the target genes were performed by the 2^−ΔΔCt^ method. Student's *t*-test was used to analyze the statistical difference by GraphPad Prism 5 (GraphPad Software Inc., San Diego, CA, USA). *P* < 0.05 and <0.01 were considered statistically significant and highly significant, respectively.

## Results

### Proliferation Characteristics of Novel Duck Reovirus in Duck Embryo Fibroblast Cells

**Figure 1:**
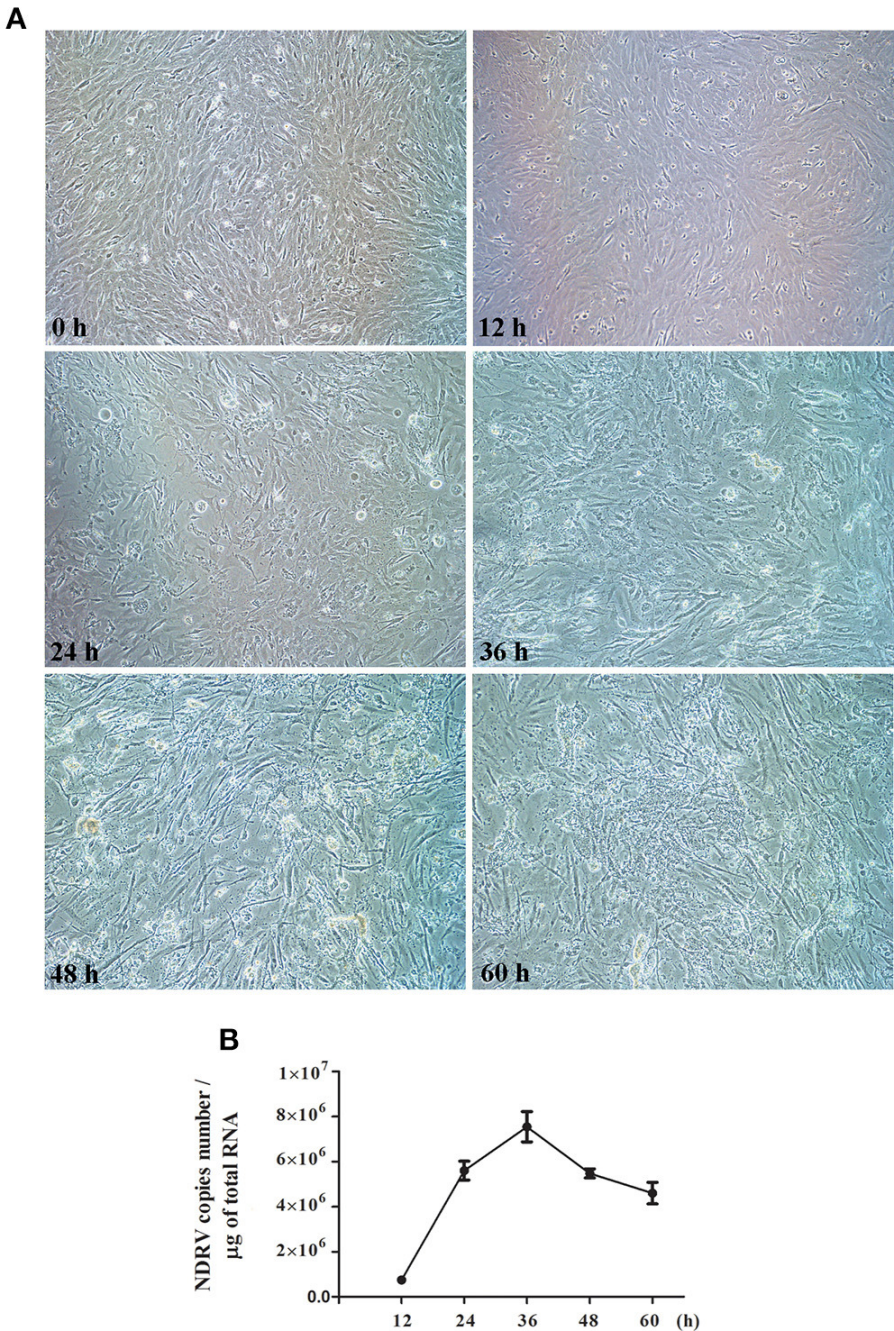
Novel duck reovirus (NDRV) infection in duck embryo fibroblast (DEF) cells. **(A)** The cytopathic effect (CPE) of DEF cells after NDRV infection. The cells were infected with NDRV at a dose of 2 × 10^2.6^ TCID_50_ per well and then observed by microscopy at the different time points. **(B)** One-step growth curve of NDRV. The infected cells were collected, and the virus titer was measured by the qPCR method.

To determine the replication kinetics of NDRV in DEF cells and the optimal time point for proteomic analysis. DEF cells cultured in six-well-plates were infected with NDRV and observed at 12, 24, 36, 48, and 60 hpi. As shown in Figure 1A, the cells infected with NDRV began to show a slight CPE at 24 hpi, and the CPE gradually became apparent with the extension of infection time. At 36 hpi, cells showed significant lesions and some cells started atrophying and shedding. Large-area shedding of the infected cells was observed at 48 and 60 hpi. These cells were collected for generating the growth curve of the NDRV in DEF cells through the qPCR method. The results showed that the titer of NDRV rapidly increased after 12 hpi and reached a peak after 36 h of infection (7.5 × 10^6^ copies/μg) and then decreased (Figure 1B). Generally, the optimal samples for proteomic analysis are during the high virus titer with the absence of severe cytoskeletonal and membrane rearrangement of the infected cells. Therefore, based on the CPE and the growth curve analyses of NDRV, the cell samples at 24 hpi were selected and processed for proteomic analysis in this study.

### Identification of Differentially Expressed Cellular Proteins

A total of 6,137 proteins were identified in cell samples at 24 hpi, according to the filter criteria of 1% FDR using the iTRAQ-coupled LC-MS/MS analysis. In the current study, we set the cutoff threshold to a 50% change in ratios and statistically significant differences at *P* < 0.05 for biological significance. Thus, NDRV-to-negative control ratios of >1.5 or <0.67 were considered to indicate “upregulation” or “downregulation.” Therefore, 179 DEPs were identified and quantified. Of these proteins, 89 and 90 proteins were upregulated or downregulated, respectively (Supplementary Tables 1, 2).

### The Functional Analysis of Differentially Expressed Cellular Proteins

**Figure 2:**
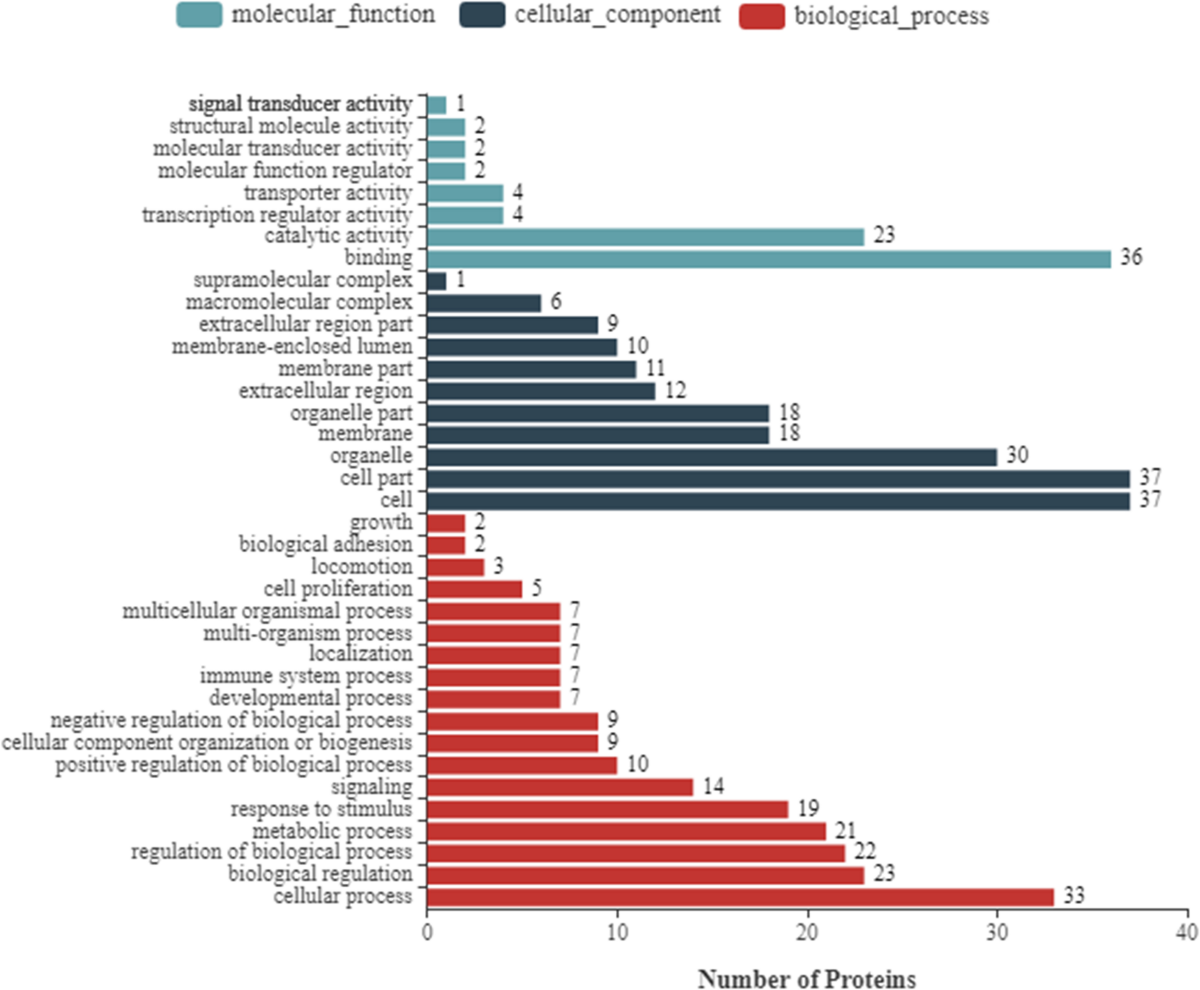
Gene Ontology (GO) annotation classification of differentially expressed proteins of NDRV infection. It consists of three different sets: molecular function, cellular component, and biological process.

**Figure 3:**
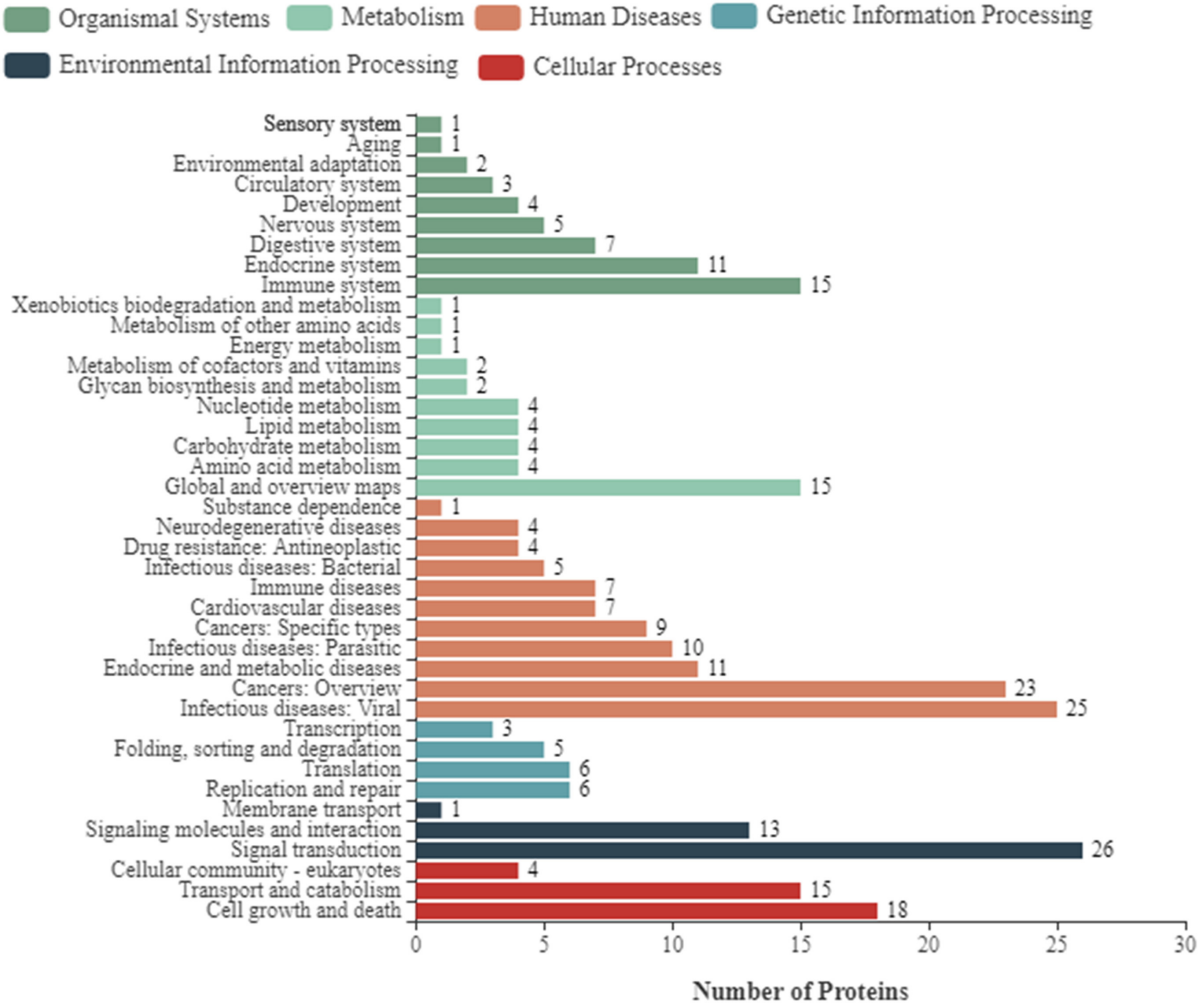
Kyoto Encyclopedia of Genes and Genomes (KEGG) pathway clustering of differentially expressed proteins of NDRV infection. The pathways include organismal systems, metabolism, human diseases, genetic information processing, environmental information processing, and cellular processes.

To further enhance the understanding of the functions of DEPs, bioinformatics analyses were performed, including the GO enrichment analysis, KEGG analysis, and subcellular localization analysis. Based on GO analysis, the DEPs were divided into three categories: cellular component, molecular function, and biological process. As shown in Figure 2, in the classification of molecular functions, the significant enrichment items are catalytic activity and molecular functional binding; in the category of cell components, the significant enrichment items are cell organelles and parts. Metabolic process, biological regulation, and cellular processes were the significantly rich terms in the biological processes. A total of 290 pathway proteins were enriched through KEGG pathway enrichment analysis, including organismal systems, metabolism, human diseases, genetic information processing, environmental information processing, and cellular processes. Among them, signal transduction, infectious diseases, cell growth and death, and the immune system have more proteins (Figure 3).

**Figure 4:**
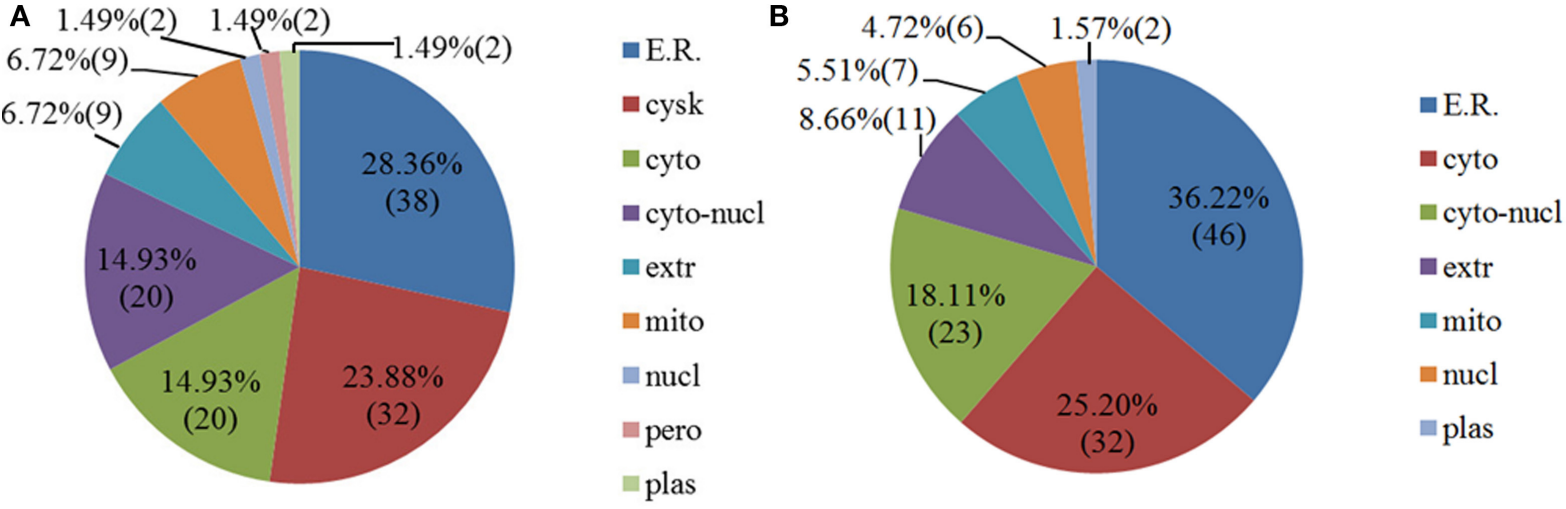
Subcellular location of differentially expressed proteins of NDRV infection. **(A)** Upregulated proteins. **(B)** Downregulated proteins. ER, endoplasmic reticulum; cysk, cytoskeleton; cyto, cytosol; cyto-nucl, cytosol-nucleus; extr, extracellular; mito, mitochondria; nucl, nucleus; pero, peroxisome; plas, plasma membrane.

Some proteins are secreted outside the cell or remain in the cytoplasm after proteins are synthesized in the ribosomes. Only when they are transported to the correct location can they participate in various physiological activities of the cell. Therefore, it is necessary to analyze the subcellular localization of DEPs. As shown in Figure 4A, the 89 upregulated proteins in NDRV-infected cells were mainly localized to the endoplasmic reticulum, cytoskeleton, cytosol, nucleus, and cytoplasm. The 90 downregulated proteins were mainly distributed in the endoplasmic reticulum, cytosol, nucleus, and cytoplasm and extracellularly (Figure 4B).

### Novel Duck Reovirus Infection Induces the Expression of Numerous Interferon-Stimulated Genes

**Figure 5:**
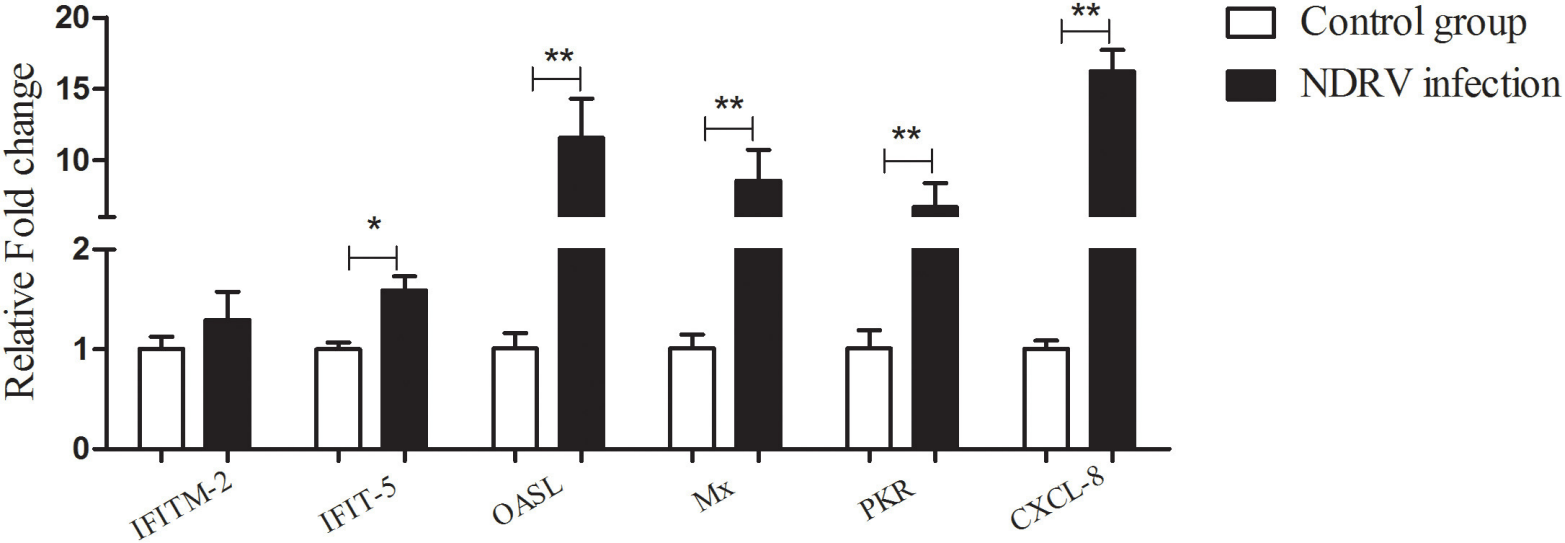
The relative expression of some differentially expressed genes in DEF cells infected with NDRV. The DEF cells cultured in six-well-plates were infected with NDRV (2 × 10^2.6^ TCID_50_/well) and collected at 24 h post infection (hpi) for the detection of immune-related genes using the qPCR method. The relative expression of genes was analyzed by the 2^−ΔΔCt^ method. *P < 0.05 was considered to be significant, and **P < 0.01 was highly significant.

According to the proteomic data, we found that a large number of ISGs were activated, such as IFN-induced transmembrane protein (IFITM)2, IFN-induced protein with tetratricopeptide repeats (IFIT)5, 2′-5′-oligoadenylate synthetase like (OASL), Mx, protein kinase R (PKR), and viperin. To confirm the results of the iTRAQ quantitative proteomic analysis, six differentially expressed genes [IFITM2, IFIT5, OASL, Mx, PKR, and C-X-C motif chemokine ligand (CXCL)-8] were selected to detect their mRNA expressions using qPCR at 24 hpi. The results showed that the expression of all selected genes was upregulated (Figure 5). For example, the mRNA transcripts of OASL and Mx increased by 11.57- and 8.57-fold, respectively, and the expression of CXCL-8 increased by 16.23-fold. Overall, the mRNA change trend of selected proteins was consistent with the quantitative results of the proteome.

## Discussion

In recent years, emerging and reemerging infectious diseases have increased in ducks and seriously threaten the healthy development of the duck industry (11, 18–22). The disease caused by NDRV is an acute and contact infectious disease in duck farms, characterized by splenomegaly, necrosis, and high mortality (6, 23). However, information about the pathogenesis of NDRV and host–virus interactions has been poorly understood. In the current study, quantitative proteomic analysis has demonstrated the profiles of DEPs in NDRV-infected DEF cells compared to the control group through iTRAQ coupled with the LC-MS/MS method, and NDRV infection activates the innate immune responses of the host cell, leading to the expression of various ISGs.

In the previous study, two-dimensional (2D) polyacrylamide gel electrophoresis (PAGE) combined with LC-MS/MS was used to identify DEPs in Muscovy DEFs infected with the different virulent MDRV strains, and 59 DEPs were identified. These DEPs had functions in the metabolism and utilization of carbohydrates and nucleotides, anti-stress, and regulation of immunity (7). In our study, the DEF cells infected with NDRV were collected for proteomic analysis at 24 dpi. We obtained relative quantitative information about 6,137 proteins, and a total of 179 DEPs were identified, of which 89 proteins were upregulated and 90 were downregulated. The results indicate that the iTRAQ method can identify more kinds and a larger number of proteins with more accuracy than by 2D electrophoresis. The DEPs induced by NDRV infection were mainly involved in molecular function, cellular component, and biological process through the GO analysis. The signaling pathways of transduction, infectious diseases, and immune system were observed according to the KEGG pathway analysis.

The correct subcellular location is essential for physiological function of DEPs. In avian reovirus (ARV)-infected DF-1 cells, the upregulated proteins were mostly located in the mitochondria (50.00%), cytoplasm (27.77%), and cytoplasm/nucleus (8.33%). The downregulated proteins were mostly located in the cytoplasm (31.03%), cytoskeleton (20.69%), and cytoplasm/nucleus (17.24%) (24). However, in our study, the upregulated DEPs were mainly located in the endoplasmic reticulum (28.36%), cytoskeleton (23.88%), cytoplasm (14.93%), and cytoplasm/nucleus (14.93%), and the downregulated proteins were mostly located in the endoplasmic reticulum (36.22%), cytoplasm (25.20%), and cytoplasm/nucleus (18.11%). The mitochondria and cytoskeleton were not the main subcellular location for DEPs in the current study, which was different from the cellular protein changes in ARV-infected cells. These results indicated that the DEPs located in different subcellular regions may have different roles in the responses to virus infections.

Autophagy is observed in viral infection, where it is involved in viral replication (25). Previous studies have proposed that ARV triggers autophagy in infected cells through activation of the p53 signaling pathway (26). In this study, the expressions of autophagy cargo receptor 1 (NBR1) and autophagy-related protein 13 were slightly upregulated (1.36- and 1.34-fold, respectively) after NDRV infection, indicating that NDRV may induce autophagy in DEF cells and affect cellular metabolism. However, the specific roles of autophagy in NDRV infection remain unclear, and further research is needed to determine whether autophagy inhibits virus replication. In addition to the autophagy-lysosome system, ubiquitin-proteasome system (UPS) is another major proteolysis system in eukaryotic cells. In ARV-infected DF-1 cells, the UPS-related proteins such as proteasome activator complex subunit (PSME) 3 and proteasome subunit a type 3 (PSMA3) were downregulated, indicating that ARV caused functional impairment of the cellular UPS to facilitate viral progeny release (24). However, in the our study, PSME 4 and several E3 ubiquitin ligases were upregulated, and proteasome inhibitor PI31 subunit was downregulated (0.69-fold). These results suggested that UPS was activated following NDRV infection, which were different from those of ARV infection. The low infection dose may contribute to this result in addition to the virus strain and cell types. In this study, the low multiplicity of infection (MOI) was used to infect ducks, which may trigger effective antiviral immune responses.

It was reported that infectious bursal disease virus could induce apoptosis in DF-1 cells through interaction with voltage-dependent anion channel (VDAC)2 (27). Several proapoptotic proteins including Bcl-2, VDAC1, and VDAC3 were upregulated following NDRV infection. However, the effect of cell apoptosis on viral replication requires further investigation. Meanwhile, two upregulated translation-related proteins, eukaryotic translation initiation factor 2-alpha kinase 3 (1.99-fold) and eukaryotic elongation factor (1.23-fold), were identified in this NDRV-infected DEF cells. These above results indicated that low NDRV infection may primarily alter the expression of some proteins involved in immune response.

More recently, comparative proteomic analysis between classical reovirus infection and NDRV infection in Muscovy ducks was conducted. Numerous DEPs were identified in the liver and spleen. Only a small number of common DEPs were identified in both liver and spleen cells, indicating that the host response patterns to the reovirus infection may be tissue-dependent. Among them, the serine proteases, including the complement, coagulation, and fibrinolytic systems, were activated in both organs, indicating that serine protease-mediated innate immune responses play an important role in *in vivo* classical reovirus and NDRV infections (9, 10). However, the DEPs belonging to the serine protease system were not observed in NDRV-infected DEF cells. We further analyzed the proteomic data of *in vivo* reovirus infections and, interestingly, found that classical reovirus and NDRV infections significantly upregulated the expression of RIG-I, MDA5, IRF3, and several ISGs, such as Mx, viperin, IFIT5, and IFITM2 in both the liver and spleen. Additionally, Toll-like receptor (TLR)3 and nuclear factor (NF)-κB in the liver and TNF receptor-associated factor (TRAF)3, IFN regulatory transcription factor (IRF)3, and IFIT5 in the spleen were also upregulated (9, 10). These results suggested that classical and NDRV infections could activate the RIG-I-like receptor (RLR) and TLR3 signaling pathway and promote the production of various ISGs, leading to the establishment of innate immune responses in infected Muscovy ducks. Furthermore, spleen transcriptome profiles of duck infected with MDRV showed that RIG-I and TLR signaling pathways, IFN-α and IFN-γ were implicated in the innate immune response to this virus (8). These DEPs involved in host innate immune responses have also been observed in our study. A qPCR verification assay confirmed that the mRNA expressions of IFITM2, IFIT5, OASL, Mx, PKR, and CXCL-8 were all upregulated, which was consistent with proteomic data. These results support our previous study that NDRV infection initiates the innate immune response and induces the expression of antiviral genes in ducklings (6). It was worth noting that the secreted upregulated type I IFN was not found on the protein level. Similar results were also observed in reovirus-infected Muscovy ducks. MDRV and NDRV infections did not upregulate the expression of type I IFN *in vivo* according to proteomic analyses. Meanwhile, several proteomic studies performed *in vitro* demonstrated that there was an abundance of ISG expression but no significant upregulation of type I IFN. Zhang et al. (28) showed that Japanese encephalitis virus infection upregulated IFIT1, IFITM1, IFITM3, ISG15, and Mx, but the upregulated type I IFN was not found based on proteomic analysis; a similar result was observed in quantitative proteomic analysis of transmissible gastroenteritis virus (29). However, it was interesting that the expression of type I IFN was induced following virus infections on transcription levels. The transcriptome profile of Muscovy ducks in response to MDRV infection identified many differentially expressed genes; IFN-α was upregulated in spleen (57.68-fold) (8). Moreover, it also found that NDRV infection can upregulate mRNA expression of type I IFN and ISGs in liver and spleen in our previous study (6). Taken together, these studies indicated that there are some differences between *in vitro* and *in vivo*; the results from proteomics and transcriptomics are not entirely consistent. We speculated that the possible reasons are as follows: (1) the different virus strains with different virulence may contribute to this discrepancy. It was reported that the mammalian orthoreovirus strain Dearing (T3D) for serotype 3 could induce significant upregulation of several proteins in the signaling pathway, including Mx1, ISG15, IFIT1, signal transducer and activator of transcription (STAT)1, IFIT3, and DExD/H-box helicase (DDX)58. However, the strain 1 Lang (T1L) for serotype 1 cannot (30); (2) infection dose. The low infection dose may primarily cause the host's antiviral immune responses; (3) compared with experiments *in vitro*, the studies *in vivo* are very complex, which may lead to different results due to individual differences in animals. Of particular interest in our study was the upregulated expression of STAT1 protein in NDRV-infected DEF cells. As it is well-known, STAT1 is an important transcription protein, and it triggers a large amount of ISG production by mediating the Janus kinase (JAK)-STAT signaling pathway. Transmissible gastroenteritis virus can also upregulate and phosphorylate STAT1 protein, resulting in the activation of the JAK-STAT1 signaling pathway (29). It has been reported that the STAT1-deficient mice were more susceptible to severe acute respiratory syndrome (SARS) and other viral diseases and displayed more serious lesions (31, 32). Therefore, we speculated that NDRV infection can efficiently activate the JAK-STAT1 signaling pathway, and STAT1 protein plays an important role in the innate immune response to NDRV infection. In a future study, the phosphorylation and nuclear accumulation of the STAT1 protein will be explored to confirm the activation of this pathway.

In summary, the proteomic changes of NDRV-infected DEF cells were characterized by iTRAQ coupled with LC-MS/MS. Notably, NDRV infection can elicit significant changes of cellular proteins and a large expression of ISGs. However, further functional studies are needed to understand the molecular response mechanism of host cells to NDRV infection.

## Data Availability Statement

All proteomics datasets have been deposited in ProteomeXchange, the accession is PXD020171.

## Ethics Statement

The animal study was reviewed and approved by the Committee on the Animal Ethics of Shandong Agricultural University.

## Author Contributions

YY, LL, and XLi wrote the article. MJ, JZ, and XLiu analyzed the proteomic data. CZ and HY made the figures. SL and NL designed and reviewed the article. All authors contributed to the article and approved the submitted version.

## Conflict of Interest

The authors declare that the research was conducted in the absence of any commercial or financial relationships that could be construed as a potential conflict of interest.
