# Supplementary material for: Quantitative Proteomic Analysis of Duck Embryo Fibroblasts Infected With Novel Duck Reovirus
Source: Front Vet Sci. 2020 Dec 2;7:577370. doi: 10.3389/fvets.2020.577370 (PMC7738351; doi:10.3389/fvets.2020.577370)
Supplement: Supplementary file 1 [file Table_1.docx]

Supplementary Table 1. The upregulated differentially expressed proteins identified in NDRV-infected cells

| NCBInr Accession | NCBInr Description | Ratio  (infection/control) | SD | Mass  (kDa) | sequence coverage(%) | peptides | Q-value < 0.05)*P* value |
| --- | --- | --- | --- | --- | --- | --- | --- |
| EOB04209.1 | Rho guanine nucleotide exchange factor 3 | 7.91 | 1.14 | 56.52 | 0.013 | 1 | 7.68E-11 |
| EOB01303.1 | hypothetical protein Anapl_03994 | 6.81 | 2.08 | 42.80 | 0.013 | 1 | 4.75E-08 |
| EOA98722.1 | Protein FAM65B, | 4.56 | 0.83 | 120.20 | 0.021 | 1 | 1.01E-08 |
| NP_001297714.1 | interferon-induced transmembrane protein 2 | 3.98 | 0.51 | 12.21 | 0.202 | 2 | 9.55E-10 |
| EOB00647.1 | E3 ubiquitin-protein ligase RNF128 | 3.86 | 2.42 | 39.35 | 0.031 | 1 | 3.99E-04 |
| APW85809.1 | 2',5'-oligoadenylate synthetase-like protein | 3.44 | 0.26 | 57.98 | 0.233 | 8 | 2.82E-11 |
| XP_012949292.1 | protein very KIND | 3.39 | 0.58 | 65.72 | 0.012 | 1 | 1.77E-08 |
| XP_005020045.1 | pyruvate dehydrogenase [acetyl-transferring]-phosphatase 2, mitochondrial | 3.09 | 0.31 | 61.26 | 0.028 | 1 | 6.47E-10 |
| EOB02040.1 | Sorting nexin-29 | 2.93 | 0.33 | 51.20 | 0.032 | 1 | 3.59E-09 |
| EOB00171.1 | Sideroflexin-4 | 2.77 | 2.28 | 21.60 | 0.045 | 1 | 3.04E-02 |
| AFH75050.1 | MHC class I antigen | 2.76 | 1.19 | 10.25 | 0.149 | 1 | 1.17E-04 |
| XP_021134540.1 | AN1-type zinc finger protein 3 isoform X3 | 2.71 | 0.66 | 21.77 | 0.074 | 1 | 2.81E-06 |
| AHK23066.1 | interferon-induced protein with tetratricopeptide repeats 5 | 2.65 | 0.27 | 56.41 | 0.067 | 3 | 2.17E-09 |
| XP_005017950.1 | metalloreductase STEAP4 | 2.62 | 0.36 | 45.91 | 0.032 | 1 | 2.46E-08 |
| EOA93083.1 | Uncharacterized protein C13orf3 | 2.51 | 0.29 | 41.49 | 0.025 | 1 | 8.09E-09 |
| EOA98746.1 | Inositol 1,4,5-trisphosphate receptor type 2 | 2.48 | 0.58 | 310.27 | 0.013 | 2 | 4.30E-06 |
| XP_005009988.1 | ubl carboxyl-terminal hydrolase 18 | 2.46 | 0.31 | 44.37 | 0.064 | 2 | 2.70E-08 |
| XP_005019653.1 | leucine-rich repeat-containing protein 31 | 2.39 | 0.31 | 64.87 | 0.017 | 1 | 5.05E-08 |
| EOA98062.1 | Centromere protein O | 2.38 | 0.78 | 31.14 | 0.052 | 1 | 1.45E-04 |
| EOA99876.1 | Poly [ADP-ribose] polymerase 12 | 2.34 | 0.15 | 87.85 | 0.053 | 4 | 2.92E-10 |
| EOB05369.1 | cAMP-specific 3',5'-cyclic phosphodiesterase 7B | 2.28 | 0.33 | 49.51 | 0.033 | 1 | 1.85E-07 |
| EOB03245.1 | Beta-1,4-galactosyltransferase 1 | 2.26 | 1.25 | 30.54 | 0.031 | 1 | 3.41E-03 |
| NP_001297349.1 | interleukin-8 precursor | 2.21 | 0.47 | 8.53 | 0.297 | 2 | 3.74E-06 |
| EOA97893.1 | Sequestosome-1 | 2.17 | 0.21 | 40.15 | 0.227 | 4 | 7.83E-09 |
| EOB05620.1 | Putative helicase MOV-10 | 2.10 | 0.39 | 94.16 | 0.134 | 1 | 3.45E-06 |
| EOA96635.1 | Transmembrane and TPR repeat-containing protein 1 | 2.04 | 0.27 | 72.67 | 0.016 | 1 | 2.81E-07 |
| EOB01083.1 | Protein cereblon | 2.00 | 0.52 | 49.71 | 0.038 | 1 | 5.19E-05 |
| EOA93226.1 | Cartilage intermediate layer protein 2 | 2.00 | 0.25 | 46.97 | 0.095 | 3 | 1.77E-07 |
| EOA99255.1 | Arginase, non-hepatic 1 | 2.00 | 0.20 | 32.31 | 0.089 | 2 | 2.52E-08 |
| ALF04449.1 | double-stranded RNA-dependent protein kinase | 1.99 | 0.09 | 56.09 | 0.112 | 5 | 5.73E-11 |
| EOA99356.1 | Inter-alpha-trypsin inhibitor heavy chain H5 | 1.98 | 0.12 | 41.07 | 0.019 | 1 | 6.17E-10 |
| XP_021134041.1 | E3 ubiquitin-protein ligase RNF213 isoform X2 | 1.97 | 0.25 | 76.33 | 0.017 | 1 | 3.82E-07 |
| XP_005031858.2 | cis-aconitate decarboxylase | 1.97 | 0.18 | 42.42 | 0.112 | 3 | 1.38E-08 |
| EOB01990.1 | Radical S-adenosyl methionine domain-containing protein 2 | 1.97 | 0.39 | 33.91 | 0.15 | 4 | 1.24E-05 |
| EOB07649.1 | Sterile alpha motif domain-containing protein 9-like | 1.96 | 0.10 | 185.53 | 0.018 | 2 | 2.17E-10 |
| EOB01912.1 | Vascular cell adhesion protein 1 | 1.95 | 0.22 | 58.55 | 0.036 | 2 | 1.14E-07 |
| EOB04545.1 | Tripartite motif-containing protein 13 | 1.94 | 0.15 | 47.68 | 0.025 | 1 | 7.16E-09 |
| EOB07150.1 | Lipase maturation factor 1 | 1.92 | 0.28 | 40.40 | 0.061 | 2 | 1.11E-06 |
| EOA95652.1 | hypothetical protein Anapl_12067 | 1.91 | 0.38 | 56.78 | 0.024 | 1 | 1.09E-05 |
| AFH74995.1 | MHC class I antigen | 1.91 | 0.26 | 10.27 | 0.149 | 1 | 4.21E-07 |
| EOB02537.1 | hypothetical protein Anapl_11596 | 1.91 | 0.73 | 29.12 | 0.023 | 1 | 3.97E-03 |
| XP_012948210.1 | E3 ubiquitin/ISG15 ligase TRIM25 isoform X3 | 1.90 | 0.17 | 71.41 | 0.115 | 7 | 3.65E-08 |
| EOB07275.1 | Signal transducer and activator of transcription 1-alpha/beta | 1.87 | 0.08 | 11.76 | 0.471 | 5 | 7.83E-11 |
| XP_005028910.1 | bcl-2-related ovarian killer protein isoform X1 | 1.85 | 0.34 | 23.91 | 0.047 | 1 | 7.38E-06 |
| XP_005024129.1 | glycerophosphodiester phosphodiesterase domain-containing protein 4 | 1.85 | 0.13 | 47.13 | 0.024 | 1 | 5.61E-09 |
| EOB01794.1 | hypothetical protein Anapl_01525 | 1.83 | 0.17 | 83.88 | 0.011 | 1 | 5.71E-08 |
| XP_021124344.1 | endonuclease/exonuclease/phosphatase family domain-containing protein 1 isoform X2 | 1.82 | 0.35 | 62.57 | 0.027 | 1 | 1.90E-05 |
| EOA93288.1 | Rho-related GTP-binding protein RhoV | 1.81 | 0.18 | 23.70 | 0.053 | 1 | 9.63E-08 |
| XP_021126766.1 | LOW QUALITY PROTEIN: G-protein coupled receptor 98 | 1.80 | 0.15 | 302.84 | 0.006 | 1 | 2.52E-08 |
| EOB06095.1 | CMP-sialic acid transporter | 1.78 | 0.57 | 31.70 | 0.025 | 1 | 8.09E-04 |
| XP_021134041.1 | E3 ubiquitin-protein ligase RNF213 isoform X2 | 1.74 | 0.05 | 489.44 | 0.045 | 18 | 5.33E-12 |
| XP_012957771.2 | COP9 signalosome complex subunit 7b | 1.74 | 0.78 | 25.42 | 0.041 | 1 | 1.35E-02 |
| XP_012964469.1 | laminin subunit alpha-4 | 1.73 | 0.30 | 183.87 | 0.006 | 1 | 1.15E-05 |
| XP_005018399.1 | periostin isoform X5 | 1.73 | 0.41 | 92.91 | 0.262 | 1 | 2.05E-04 |
| XP_012959616.2 | epithelial-stromal interaction protein 1 | 1.72 | 0.19 | 32.46 | 0.026 | 1 | 4.82E-07 |
| EOB00590.1 | Poly [ADP-ribose] polymerase 9 | 1.71 | 0.20 | 86.08 | 0.04 | 3 | 9.73E-07 |
| XP_005024317.1 | fatty acid desaturase 2 | 1.71 | 0.16 | 52.20 | 0.124 | 5 | 1.51E-07 |
| EOA94414.1 | Tumor necrosis factor receptor superfamily member 6 | 1.70 | 0.12 | 40.74 | 0.032 | 1 | 1.39E-08 |
| XP_005016825.1 | complement C1s subcomponent | 1.70 | 0.25 | 79.16 | 0.071 | 4 | 6.14E-06 |
| XP_005024936.1 | uncharacterized protein LOC101804575 | 1.68 | 0.11 | 34.06 | 0.045 | 1 | 8.81E-09 |
| EOA98550.1 | Eukaryotic translation initiation factor 2-alpha kinase 3 | 1.66 | 0.12 | 114.99 | 0.011 | 1 | 2.97E-08 |
| EOA96734.1 | Interferon-induced GTP-binding protein Mx | 1.66 | 0.53 | 71.49 | 0.037 | 2 | 5.85E-03 |
| EOB03540.1 | Fibulin-2 | 1.66 | 0.13 | 138.75 | 0.142 | 15 | 4.19E-08 |
| EOA99252.1 | Zinc transporter ZIP9 | 1.65 | 0.24 | 28.93 | 0.055 | 1 | 7.02E-06 |
| XP_005021559.1 | ras-related protein Rab-19 isoform X2 | 1.64 | 0.25 | 24.30 | 0.051 | 1 | 1.71E-05 |
| EOB00236.1 | Nitric oxide synthase, inducible | 1.62 | 0.10 | 128.30 | 0.027 | 3 | 1.46E-08 |
| XP_005010095.1 | dual specificity protein phosphatase 16 | 1.60 | 0.14 | 74.36 | 0.011 | 1 | 2.38E-07 |
| XP_005019348.1 | angiopoietin-related protein 1 | 1.60 | 0.27 | 57.04 | 0.026 | 1 | 3.38E-05 |
| XP_005030968.1 | tumor necrosis factor alpha-induced protein 2 | 1.60 | 0.09 | 61.23 | 0.141 | 7 | 6.52E-09 |
| XP_005029143.1 | ectoderm-neural cortex protein 1-like | 1.59 | 0.20 | 66.66 | 0.008 | 1 | 2.80E-06 |
| XP_021130696.1 | LOW QUALITY PROTEIN: uncharacterized protein LOC101799256 | 1.58 | 0.21 | 126.42 | 0.006 | 1 | 5.94E-06 |
| XP_005028887.1 | leucine-rich repeat and fibronectin type III domain-containing protein 1-like protein isoform X2 | 1.58 | 0.15 | 66.74 | 0.02 | 1 | 6.09E-07 |
| XP_005012854.1 | rho GTPase-activating protein 24 isoform X2 | 1.57 | 0.37 | 73.95 | 0.02 | 1 | 5.47E-04 |
| EOB05097.1 | Actin filament-associated protein 1-like 2 | 1.57 | 0.27 | 93.73 | 0.01 | 1 | 4.88E-05 |
| EOA95520.1 | Basement membrane-specific heparan sulfate proteoglycan core protein | 1.57 | 0.09 | 19.67 | 0.2 | 3 | 1.26E-08 |
| XP_005009112.1 | ras-related protein Rab-20 | 1.56 | 0.18 | 25.94 | 0.048 | 1 | 2.61E-06 |
| EOA97294.1 | Acyl-CoA desaturase | 1.55 | 0.05 | 41.08 | 0.148 | 6 | 1.30E-10 |
| EOA99178.1 | Protein YIPF5 | 1.55 | 0.16 | 24.25 | 0.041 | 1 | 1.40E-06 |
| EOB08882.1 | Bloom syndrome protein-like protein | 1.55 | 0.39 | 127.61 | 0.009 | 1 | 8.61E-04 |
| XP_005019138.1 | caspase recruitment domain-containing protein 19 isoform X4 | 1.54 | 0.41 | 19.98 | 0.144 | 2 | 1.44E-03 |
| EOB00583.1 | Poly [ADP-ribose] polymerase 14 | 1.54 | 0.10 | 203.05 | 0.099 | 13 | 4.54E-08 |
| EOB06262.1 | Tetratricopeptide repeat protein 17 | 1.53 | 0.59 | 125.72 | 0.016 | 2 | 3.98E-02 |
| EOB01995.1 | UMP-CMP kinase 2, mitochondrial | 1.53 | 0.29 | 16.63 | 0.107 | 1 | 1.36E-04 |
| ADG04697.1 | CENP-U, partial [Anas platyrhynchos] | 1.52 | 0.14 | 26.47 | 0.065 | 1 | 8.10E-07 |
| XP_005031920.1 | LOW QUALITY PROTEIN: acylpyruvase FAHD1, mitochondrial | 1.52 | 0.26 | 25.60 | 0.096 | 1 | 1.29E-04 |
| EOB07152.1 | B9 domain-containing protein 1 | 1.51 | 0.32 | 20.87 | 0.071 | 1 | 3.74E-04 |
| EOB00006.1 | COMM domain-containing protein 4 | 1.51 | 0.08 | 17.45 | 0.145 | 1 | 2.14E-08 |
| XP_005023911.1 | helicase ARIP4 isoform X1 | 1.51 | 0.14 | 158.63 | 0.009 | 1 | 1.22E-06 |
| EOA97890.1 | Heterogeneous nuclear ribonucleoprotein H | 1.51 | 0.28 | 53.48 | 0.391 | 1 | 1.63E-04 |
