# Supplementary material for: Quantitative Proteomic Analysis of Duck Embryo Fibroblasts Infected With Novel Duck Reovirus
Source: Front Vet Sci. 2020 Dec 2;7:577370. doi: 10.3389/fvets.2020.577370 (PMC7738351; doi:10.3389/fvets.2020.577370)
Supplement: Supplementary file 2 [file Table_2.docx]

Supplementary Table 2. The upregulated differentially expressed proteins identified in NDRV-infected cells

| NCBInr Accession | NCBInr Description | Ratio  (infection/control) | SD | Mass  (kDa) | sequence coverage(%) | peptides | Q-value < 0.05)*P* value |
| --- | --- | --- | --- | --- | --- | --- | --- |
| XP_021127319.1 | acyl-coenzyme A synthetase ACSM4, mitochondrial-like isoform X2 | 0.16 | 0.05 | 61.00 | 0.045 | 1 | 1.36E-07 |
| XP_005019005.1 | kinetochore protein Spc25 | 0.26 | 0.03 | 31.35 | 0.056 | 1 | 1.11E-09 |
| XP_021124014.1 | PGAP2-interacting protein | 0.33 | 0.03 | 73.24 | 0.026 | 1 | 1.43E-10 |
| EOA92794.1 | hypothetical protein Anapl_19132 | 0.36 | 0.06 | 19.97 | 0.071 | 1 | 5.38E-08 |
| EOB03390.1 | hypothetical protein Anapl_12121 | 0.36 | 0.05 | 68.79 | 0.018 | 1 | 1.65E-08 |
| XP_012949920.1 | anillin isoform X1 | 0.37 | 0.05 | 120.80 | 0.042 | 4 | 1.84E-08 |
| EOB07707.1 | Delta and Notch-like epidermal growth factor-related receptor | 0.38 | 0.04 | 73.49 | 0.017 | 1 | 2.58E-09 |
| XP_005025437.1 | serine/threonine-protein phosphatase 2A regulatory subunit B'' subunit beta isoform X1 | 0.38 | 0.09 | 67.92 | 0.023 | 1 | 2.94E-06 |
| XP_012954082.1 | heterogeneous nuclear ribonucleoprotein M isoform X1 | 0.41 | 0.08 | 66.10 | 0.434 | 1 | 1.11E-06 |
| XP_005030342.1 | cdc42 effector protein 3 | 0.42 | 0.09 | 27.97 | 0.063 | 1 | 1.91E-06 |
| XP_012954653.1 | cAMP-regulated phosphoprotein 21 isoform X14 | 0.44 | 0.05 | 82.57 | 0.011 | 1 | 2.23E-08 |
| XP_021125994.1 | LOW QUALITY PROTEIN: DNA topoisomerase 2-binding protein 1-like | 0.46 | 0.06 | 129.84 | 0.011 | 1 | 1.19E-07 |
| XP_005009848.2 | thrombospondin-1 | 0.46 | 0.03 | 130.72 | 0.253 | 27 | 1.16E-10 |
| XP_005031007.1 | ubiquitin-like protein fubi and ribosomal protein S30 | 0.46 | 0.09 | 14.44 | 0.083 | 2 | 1.74E-06 |
| EOB00318.1 | Fanconi anemia group D2 protein | 0.46 | 0.07 | 155.79 | 0.013 | 1 | 1.48E-07 |
| EOB00061.1 | Heterogeneous nuclear ribonucleoprotein K | 0.47 | 0.21 | 45.60 | 0.243 | 1 | 1.32E-03 |
| EOA95097.1 | Protein SPT2-like protein | 0.47 | 0.06 | 67.79 | 0.039 | 2 | 8.70E-08 |
| EOB03157.1 | Membralin | 0.48 | 0.08 | 55.11 | 0.019 | 1 | 1.10E-06 |
| XP_012948530.1 | HEAT repeat-containing protein 6 | 0.49 | 0.20 | 122.37 | 0.025 | 2 | 3.04E-04 |
| XP_005012371.1 | importin subunit alpha-1 | 0.49 | 0.02 | 58.40 | 0.178 | 8 | 2.55E-11 |
| XP_012957632.1 | protein CYR61 | 0.5 | 0.06 | 36.42 | 0.294 | 8 | 2.39E-07 |
| XP_021135432.1 | deoxynucleotidyltransferase terminal-interacting protein 2-like | 0.5 | 0.08 | 57.38 | 0.089 | 1 | 9.53E-07 |
| EOA99102.1 | Ribonucleoside-diphosphate reductase large subunit | 0.51 | 0.04 | 88.11 | 0.116 | 4 | 9.37E-09 |
| XP_005027616.1 | pentraxin-related protein PTX3 | 0.51 | 0.06 | 44.15 | 0.357 | 13 | 7.26E-08 |
| EOB00784.1 | Protein regulator of cytokinesis 1 | 0.52 | 0.07 | 60.03 | 0.043 | 2 | 4.85E-07 |
| XP_005026782.1 | transferrin receptor protein 1 | 0.52 | 0.04 | 86.43 | 0.321 | 18 | 1.05E-08 |
| EOA95941.1 | Ankyrin repeat domain-containing protein 52 | 0.52 | 0.08 | 116.60 | 0.019 | 1 | 9.18E-07 |
| EOA92910.1 | DNA replication licensing factor MCM7 | 0.52 | 0.04 | 9.37 | 0.143 | 1 | 1.18E-08 |
| XP_012959393.1 | protein Mdm4 | 0.53 | 0.14 | 52.00 | 0.018 | 1 | 5.62E-05 |
| XP_005027021.1 | kinesin-like protein KIF20A | 0.53 | 0.05 | 92.59 | 0.023 | 2 | 4.01E-08 |
| EOA97853.1 | Translin-associated protein X | 0.53 | 0.05 | 32.57 | 0.049 | 1 | 4.45E-08 |
| XP_021127883.1 | mitotic checkpoint serine/threonine-protein kinase BUB1 beta | 0.54 | 0.11 | 122.34 | 0.007 | 1 | 1.65E-05 |
| EOA97223.1 | Inter-alpha-trypsin inhibitor heavy chain H4 | 0.55 | 0.05 | 23.71 | 0.047 | 1 | 7.01E-08 |
| EOA94903.1 | UPF0368 protein Cxorf26 | 0.55 | 0.04 | 10.21 | 0.23 | 2 | 3.72E-09 |
| EOA93166.1 | Ubiquitin-conjugating enzyme E2 R1 | 0.55 | 0.05 | 18.27 | 0.08 | 1 | 3.75E-08 |
| EOA94643.1 | Uncharacterized protein C19orf45 | 0.55 | 0.06 | 49.34 | 0.032 | 1 | 1.41E-07 |
| EOA96046.1 | mTERF domain-containing protein 1, mitochondrial | 0.55 | 0.10 | 35.81 | 0.035 | 1 | 7.22E-06 |
| XP_005016659.1 | LOW QUALITY PROTEIN: cationic amino acid transporter 3 | 0.56 | 0.03 | 63.64 | 0.022 | 1 | 2.30E-09 |
| EOA95183.1 | YrdC domain-containing protein, mitochondrial | 0.56 | 0.02 | 18.95 | 0.07 | 1 | 5.28E-11 |
| EOA98060.1 | Transmembrane protein C2orf18-like protein | 0.56 | 0.07 | 33.49 | 0.078 | 2 | 7.71E-07 |
| XP_021130846.1 | ryanodine receptor 2 | 0.57 | 0.16 | 556.50 | 0.002 | 1 | 5.12E-04 |
| EOA95553.1 | Coiled-coil domain-containing protein 49 | 0.57 | 0.09 | 48.36 | 0.034 | 1 | 7.68E-06 |
| EOA95597.1 | E3 ubiquitin-protein ligase UHRF1 | 0.58 | 0.06 | 84.02 | 0.081 | 6 | 3.55E-07 |
| XP_005011437.1 | cellular nucleic acid-binding protein isoform X4 | 0.58 | 0.10 | 20.07 | 0.171 | 3 | 1.21E-05 |
| EOA99302.1 | Deoxyribonuclease-2-beta | 0.58 | 0.05 | 42.08 | 0.065 | 1 | 1.10E-07 |
| EOA98645.1 | Uncharacterized protein C10orf88 | 0.58 | 0.06 | 33.55 | 0.043 | 1 | 1.83E-07 |
| XP_021130005.1 | deoxyribonuclease-2-beta | 0.58 | 0.15 | 38.70 | 0.071 | 1 | 1.82E-04 |
| XP_005012179.1 | coiled-coil-helix-coiled-coil-helix domain-containing protein 7 | 0.58 | 0.03 | 10.50 | 0.333 | 4 | 2.54E-09 |
| EOA96002.1 | E3 ubiquitin-protein ligase CBL | 0.59 | 0.09 | 90.58 | 0.022 | 2 | 3.83E-06 |
| EOA92892.1 | Serine/threonine-protein phosphatase 5 | 0.59 | 0.13 | 6.96 | 0.25 | 1 | 6.70E-05 |
| XP_012964785.1 | uncharacterized protein LOC101805095 | 0.6 | 0.02 | 32.48 | 0.111 | 2 | 8.35E-11 |
| EOA96291.1 | TP53-regulated inhibitor of apoptosis 1 | 0.6 | 0.13 | 5.31 | 0.562 | 2 | 4.54E-05 |
| XP_005012842.1 | AF4/FMR2 family member 1 isoform X4 | 0.6 | 0.23 | 125.76 | 0.008 | 1 | 3.48E-03 |
| XP_012960316.1 | nucleolar GTP-binding protein 2 | 0.61 | 0.09 | 69.46 | 0.101 | 5 | 6.47E-06 |
| EOA94736.1 | Hyaluronan mediated motility receptor | 0.61 | 0.04 | 72.75 | 0.019 | 1 | 2.05E-08 |
| EOA99320.1 | Zinc finger HIT domain-containing protein 6 | 0.61 | 0.03 | 28.70 | 0.025 | 1 | 2.30E-09 |
| EOA95851.1 | Follistatin | 0.61 | 0.05 | 28.85 | 0.173 | 4 | 1.49E-07 |
| XP_005021621.1 | rac GTPase-activating protein 1 | 0.61 | 0.12 | 67.10 | 0.02 | 1 | 6.30E-05 |
| XP_005024572.2 | mucin-5AC | 0.61 | 0.09 | 218.12 | 0.004 | 1 | 6.53E-06 |
| EOB01147.1 | Acetylcholinesterase collagenic tail peptide | 0.62 | 0.09 | 24.31 | 0.036 | 1 | 9.17E-06 |
| XP_021132793.1 | LOW QUALITY PROTEIN: uncharacterized protein LOC101798158 | 0.62 | 0.08 | 27.98 | 0.097 | 2 | 4.49E-06 |
| XP_005028467.1 | general transcription factor IIH subunit 1 isoform X1 | 0.63 | 0.03 | 62.30 | 0.038 | 2 | 3.99E-09 |
| EOA97593.1 | Transcriptional repressor p66-alpha | 0.63 | 0.11 | 9.66 | 0.159 | 1 | 3.29E-05 |
| EOB02797.1 | MKI67 FHA domain-interacting nucleolar phosphoprotein-like | 0.63 | 0.09 | 22.27 | 0.202 | 1 | 7.19E-06 |
| XP_021126454.1 | kinesin-like protein KIF15 isoform X2 | 0.63 | 0.09 | 161.51 | 0.065 | 7 | 8.17E-06 |
| EOB00869.1 | Plakophilin-2 | 0.63 | 0.02 | 83.00 | 0.027 | 2 | 2.97E-10 |
| XP_005029656.1 | tumor protein 63 isoform X4 | 0.64 | 0.10 | 66.3876 | 0.01 | 1 | 2.04E-05 |
| EOA99906.1 | Legumain | 0.64 | 0.09 | 45.49 | 0.066 | 3 | 1.36E-05 |
| EOB08353.1 | Steroid receptor RNA activator 1 | 0.64 | 0.08 | 20.63 | 0.142 | 2 | 2.95E-06 |
| XP_021122854.1 | LIM and cysteine-rich domains protein 1 | 0.64 | 0.05 | 41.20 | 0.287 | 9 | 8.13E-08 |
| EOB09113.1 | TSC22 domain family protein 1 | 0.64 | 0.12 | 88.01 | 0.012 | 1 | 1.04E-04 |
| EOA94790.1 | Collagen alpha-1(XII) chain | 0.64 | 0.03 | 337.63 | 0.284 | 72 | 4.96E-09 |
| XP_021125530.1 | LOW QUALITY PROTEIN: gamma-glutamyl hydrolase | 0.64 | 0.08 | 32.34 | 0.053 | 1 | 3.41E-06 |
| EOA97568.1 | Folate transporter 1 | 0.64 | 0.08 | 50.94 | 0.031 | 1 | 3.83E-06 |
| EOA97957.1 | Glucose-fructose oxidoreductase domain-containing protein 1 | 0.65 | 0.02 | 34.43 | 0.036 | 1 | 3.68E-10 |
| EOB08054.1 | UPF0549 protein C20orf43-like protein | 0.65 | 0.03 | 16.74 | 0.083 | 1 | 9.91E-10 |
| XP_021127321.1 | regulating synaptic membrane exocytosis protein 1 isoform X27 | 0.65 | 0.10 | 152.27 | 0.019 | 2 | 1.48E-05 |
| XP_005031648.3 | DNA topoisomerase 2-binding protein 1 | 0.65 | 0.11 | 12.60 | 0.092 | 1 | 4.66E-05 |
| EOB00546.1 | U3 small nucleolar RNA-associated protein 14-like protein A | 0.65 | 0.07 | 75.56 | 0.159 | 7 | 3.30E-06 |
| EOA99348.1 | Follistatin-related protein 1 | 0.65 | 0.03 | 35.13 | 0.221 | 5 | 6.15E-09 |
| EOA96032.1 | Syndecan-2 | 0.65 | 0.05 | 20.48 | 0.048 | 1 | 7.45E-08 |
| EOB06641.1 | Deoxycytidylate deaminase | 0.66 | 0.33 | 20.75 | 0.056 | 1 | 1.05E-02 |
| EOB06161.1 | Putative dimethyladenosine transferase | 0.66 | 0.03 | 27.98 | 0.037 | 1 | 3.64E-09 |
| EOA95352.1 | Tumor necrosis factor receptor superfamily member 6B | 0.66 | 0.17 | 33.19 | 0.025 | 1 | 1.30E-03 |
| EOA96714.1 | 60S ribosomal protein L21 | 0.66 | 0.07 | 18.60 | 0.219 | 4 | 2.43E-06 |
| EOA93849.1 | Translationally-controlled tumor protein | 0.66 | 0.05 | 15.59 | 0.232 | 3 | 3.42E-07 |
| XP_005010983.1 | cyclin-dependent kinase 6 | 0.66 | 0.03 | 37.15 | 0.025 | 1 | 2.09E-09 |
| EOB09046.1 | Collagen alpha-1(IV) chain | 0.66 | 0.08 | 112.76 | 0.022 | 2 | 5.63E-06 |
| XP_012964386.2 | LOW QUALITY PROTEIN: ATP-dependent RNA helicase DDX54 | 0.66 | 0.04 | 90.67 | 0.07 | 5 | 6.28E-08 |
| EOB03487.1 | UPF0534 protein C4orf43-like protein | 0.66 | 0.05 | 22.34 | 0.121 | 2 | 9.06E-08 |
